# Salvage total laryngectomy: is a flap necessary?^☆^

**DOI:** 10.1016/j.bjorl.2018.11.007

**Published:** 2018-12-31

**Authors:** Ricardo Gonzalez-Orús Álvarez-Morujo, Paula Martinez Pascual, Manuel Tucciarone, Mario Fernández Fernández, Rosalia Souviron Encabo, Tomás Martinez Guirado

**Affiliations:** Hospital General Universitario Gregorio Marañón, Departamento de Otorrinolaringología, Cirugía de Cabeza y Cuello, Madrid, Spain

**Keywords:** Salvage total laryngectomy, Pectoralis major flap, Pharyngocutaneous fistula, Radiotherapy, Laringectomia total de resgate, Retalho do peitoral maior, Fístula faringocutânea, Radioterapia

## Abstract

**Introduction:**

Pharyngocutaneous fistula is the most significant complication after salvage total laryngectomy in patients who have received previous treatment with radiotherapy with or without chemotherapy.

**Objective:**

Our purpose is to review the fistula rate in radiated patients undergoing salvage total laryngectomy, to determine if the use of pectoralis major flap interposition reduces the incidence and duration of fistula and to examine other risk factors.

**Methods:**

We made a retrospective review of patients undergoing salvage total laryngectomy for exclusively larynx cancer after failure of primary curative radiotherapy between 2000 and 2017. General data from patients, risk factors and other complications were analyzed.

**Results:**

We identified 27 patients whose mean age was 66.4 years, mainly male (92.5%). The primary closure group without pectoralis major flap included 14 patients, and the group with pectoralis major flap closure included 13 patients. Pharyngocutaneous fistula was present in 15 patients (55.5%). Global pharyngocutaneous fistula rate was higher in the group of patients without pectoralis major flap comparing with those were the flap was interposed (78.6% versus 30.8%, *p* = 0.047). Also the pharyngocutaneous fistulas which need to be repaired with surgery (64.3% versus 7.7%, *p* = 0.03) and large pharyngostomes (64.3% versus 0%, *p* = 0.0004) were present in a higher rate in the group closed primary without pectoralis major flap. We did not find other risk factors with statistical significance. Oral diet initiation (84 days versus 21.5 days, *p* = 0.039) and the duration of hospitalization (98.3 days versus 27.2 days, *p* = 0.0041) were much lower in patients with a preventive pectoralis major flap. Two patients died as a consequence of complications of large pharyngostomes.

**Conclusions:**

Prophylactic pectoralis major flap reduced the incidence, severity and duration of fistula and should be considered during salvage total laryngectomy.

## Resumo

**Introdução:**

A fístula faringocutânea é a complicação mais significativa após laringectomia total de resgate em pacientes que receberam tratamento prévio com radioterapia com ou sem quimioterapia.

**Objetivo:**

Revisar a taxa de fístula em pacientes irradiados submetidos a laringectomia total de resgate, para determinar se o uso de interposição de retalho do peitoral maior reduz a incidência e a duração da fístula e examinar outros fatores de risco.

**Método:**

Fizemos uma revisão retrospectiva de pacientes submetidos à laringectomia total de resgate para câncer exclusivamente laríngeo após falha da radioterapia curativa primária entre 2000 e 2017. Dados gerais dos pacientes, fatores de risco e outras complicações foram analisados.

**Resultados:**

Foram identificados 27 pacientes com média de 66,4 anos, principalmente do sexo masculino (92,5%). O grupo de fechamento primário sem retalho de peitoral maior incluiu 14 pacientes e o grupo de fechamento com retalho de peitoral maior incluiu 13 pacientes. Fístula faringocutânea esteve presente em 15 pacientes (55,5%). A taxa global de fístula faringocutânea foi maior no grupo de pacientes sem retalho de peitoral maior em comparação com aqueles que receberam o retalho (78,6% vs. 30,8%, p = 0,047). Além disso, as fístulas faringocutâneas que precisaram ser reparadas através de cirurgia (64,3% vs. 7,7%, p = 0,03) e grandes faringostomias (64,3% vs. 0%, p = 0,0004) apresentaram uma taxa mais alta no grupo fechado primariamente sem retalho do peitoral maior. Não encontramos outros fatores de risco com significância estatística. O início da dieta oral (84 dias vs. 21,5 dias, p = 0,039) e a duração da internação (98,3 dias vs. 27,2 dias, p = 0,0041) foram muito menores nos pacientes com uso preventivo do retalho do peitoral maior. Dois pacientes morreram em consequência de complicações de grandes faringostomias.

**Conclusões:**

O uso profilático do retalho do peitoral maior reduziu a incidência, a gravidade e a duração da fístula e deve ser considerado durante a laringectomia total de resgate.

## Introduction

Total laryngectomy has proven to be a highly effective therapeutic tool in the treatment of advanced stages of laryngeal cancer. However, the management of advanced laryngeal cancer has changed over the past 30 years. Due to the emergence of organ-sparing strategies the use of total laryngectomy as a primary treatment in locally advanced cancer has significantly decreased over the last two decades. Combination therapy with chemotherapy and radiation therapy allow preservation of the larynx without a reduction in disease control compared with Total Laryngectomy (TL) with adjuvant Radiation Therapy (RT), as demonstrated by The Department of Veterans Affairs (VA) Laryngeal Cancer Study.^1^ The results of the Intergroup Radiation Therapy Oncology Group 91–11 (RTOG 91–11) study contributed to validation of this organ preservation approach, and demonstrated the superiority of primary concurrent Chemoradiation Therapy (CRT) over induction chemotherapy-radiation therapy and RT alone.^2^ However, there continues to be a substantial number of patients who need Salvage Total Laryngectomy (STL), owing to the fact that organ preservation rates are between 60% and 80% at 5 years, commonly due to persistent or recurrent laryngeal cancer, second primary tumors, and occasionally for laryngeal dysfunction or radionecrosis. It is well established that postoperative complications are higher in patients undergoing isolated radiotherapy or chemo radiotherapy.^3^, ^4^

Pharyngocutaneous Fistula (PCF) is the most common complication after total laryngectomy. PCF imposes a great morbidity on patients. Among its consequences are an increase in hospitalization time, higher costs, as well as the delay in the beginning of adjuvant therapy.^5^ Chronic obstructive pulmonary disease, previous hemoglobin < 12 g/dL, blood transfusion, advanced primary tumours, supraglottic subsite, hypopharyngeal tumor site, positive surgical margins or the addition of neck dissection are some of the risk factors attributed to pharyngocutaneous fistula.^6^ Multiple studies have paid special attention to complications in wound healing of patients undergoing STL.^7^, ^8^ Pharyngocutaneous fistula incidence following primary laryngectomy can be 9–25%, but can reach 14–57% in patients requiring salvage procedures.^9^, ^10^ Wound healing in a previous irradiated neck region is often impaired because of widespread scarring, fibrotic tissue remodeling and altered perfusion of irradiated tissues.^3^, ^11^

Many surgical techniques have been suggested to prevent this complication, and several authors have reported the use of non-irradiated tissue to reinforce the pharyngeal closure. The choices of donor tissue include Pectoralis Major Myofascial Flap (PMMF), Pectoralis Major Myocutaneous Flap (PMMC), or free flaps. These techniques allow incorporation of a less compromised blood supply tissue not previously irradiated and can improve wound healing with fewer complications. Nonetheless, the efficacy and indications for these approaches is not fully established.

The purpose of this study is to review fistula rates in patients undergoing salvage total laryngectomy and to assess the impact of pectoralis mayor muscle flap use in reducing fistula frequency and severity.

## Methods

We present a retrospective review of patients with previous laryngeal cancer undergoing TL after having received definitive RT with or without chemotherapy between 2000 and 2017. The following exclusion criteria were applied: (1) patients with total or partial pharyngectomies that required an increased resection of the pharyngeal mucosa (hypopharyngeal cancers); (2) patients in whom a laryngectomy was performed in a previously radiated neck as a result of the treatment of a tumor of upper aerodigestive tract other than the larynx; (3) patient with partial surgery of the larynx who received complementary radiotherapy, experienced recurrence and underwent a salvage total laryngectomy; (4) finally, patients with a salvage partial laryngectomy who require a total laryngectomy afterwards.

We collected information regarding different variables: demographic (sex, age), clinical (location, tumour size, pathological node status, tumor stage, chemotherapy or not, time to rescue, associated diseases, anesthetic evaluation), biochemistry (haemoglobin, creatinine, total proteins), surgical (neck dissection, tracheoesophageal puncture, prior tracheostomy, pectoralis major flap), and histological (tumor grade). A minimal follow-up of 6 months after surgery was required. We classified the pharyngocutaneous fistula differentiating small punctate fistulas (<8 mm, Grade 1) from large pharyngostomes (Grades 2 or more).

### Statistical analysis

Descriptive variables were summarized by mean values (SD) for continuous variables and number (percentage) for categorical variables. Chi-squared and Fisher's exact tests were used in univariate analyses. Unpaired Student's *t*-tests were used to compare means. *p* < 0.05 values were considered statistically significant.

## Results

Out of 135 patients with TL, 34 had a prior history of radiation. We excluded 7 patients, so a total of 27 patients who underwent salvage total laryngectomy were included in this study. The mean patient age was 66.4 ± 10.3 years (range 43–84 years) and most patients were men (92.5%). Patient and tumor characteristics at diagnosis, comorbidities and prior treatment are shown in Table 1. Mean time until salvage total laryngectomy was 18.2 ± 19.04 months (range 3–104 months). 81% of patients experienced progression or recurrence in the first 24 months, and 30% in the first year. We considered two groups based on the pharyngeal closure technique (primary closure with and without pectoralis major flap). The primary closure group without Pectoralis Major Flap (PMF) included 14 patients, and the group with the PMP closure included 13 patients. In the latter, 9 patients were Pectoralis Major Myofascial Flap (PMMF) and 4 Pectoralis Major Myocutaneous Flap (PMMC). Pharyngocutaneous fistula was present in 15 patients (55.5%), 6 of them with Grade 1 fistulas (22.2%), and 9 patients (33.3%) with large pharyngostomes (Grade 2 or more). In Table 2 are shown patients and tumor characteristics and association with fistula. We did not find statistically significant differences in relation to size, node status, tumor stage, location, previous treatment (chemotherapy or not), associated diseases, or levels of haemoglobin, creatinine or proteins. Likewise we encountered no significant differences in patients with neck dissection, prior tracheostomy, or tracheoesophageal puncture. The only variable that was significant was the interposition of a pectoralis major flap. Global PCF rate was higher in the group of patients without PMP comparing with those were the flap was interposed (78.6% vs. 30.8%, *p *= 0.047). Also the PCFs which need to be repaired with surgery (64.3% vs. 7.7%, *p *= 0.03) and large pharyngostomes (64.3% vs. 0%, *p *= 0.0004) were present in a higher rate in the group with primary closure without PMP (Table 3). Seven patients developed large pharyngostomes (more than 8 mm), all of them with primary closure without PMF. All these patients needed other reconstructive procedures, with a mean of 2.4 additional surgeries. Other postoperative complications included wound infection (7.4%), pneumonia (7.4%) and urinary sepsis (3.7%). Two patients died in the postoperative period due to the complications of large fistulas (7.4%), all of them with primary closure without PMF interposition: one with an massive haemorrhage at 58 days due to rupture of the carotid artery, and another that presented several episodes of bleeding, aspiration, with subsequent death at 60 days due to aspiration pneumonia. Although mean surgical time was higher in patients where the PMP was interposed, if we consider the surgical time including also all the procedures that were necessary to repair large pharyngostomes in the group of patients without prophylactic PMP, the average surgical time of each of these patients was longer. We also observed that oral diet initiation and length of hospitalization was shorter in the group with PMF closure (Table 4).

**Table 1:** Patient ant tumour characteristics, comorbidities and prior treatment.

Characteristic	*n*	%
*Sex*		
Male	25	92.5
Female	2	7.5

*Prior treatment*		
Radiotherapy alone	13	48.1
Chemo-radiotherapy	12	44.4
Bio-radiotherapy^a^	2	7.5

*Localization*		
Supraglottis	12	44.4
Glottis	15	55.6

*Pathological tumour stage*		
T1	4	14.8
T2	7	25.9
T3	13	48.1
T4	3	11.1

*Pathological node status*		
N0	23	85.2
N1	2	7.5
N2	1	3.7
N3	1	3.7

*Tumor stage*		
I	3	11.1
II	7	25.9
III	13	48.1
IVa	3	11.1
IVb	1	3.7

*Tumour grade*		
Well differentiated	7	25.9
Moderately differentiated	16	59.3
Poorly differentiated	4	14.8

*Associated diseases*		
Coronary artery disease	4	14.8
Chronic obstructive pulmonary disease	8	29.6
Hypertension	10	37
Diabetes mellitus	8	29.6
Chronic renal failure	2	7.5

*Anesthetic assessment*		
ASA II	13	48.1
ASA III	10	37
ASA IV	4	14.8

*Prior tracheostomy*		
Yes	10	37
No	17	63

*Neck dissection*		
Yes	4	14.8
No	23	85.2

*Tracheoesophageal puncture*		
Yes	5	18.5
No	22	81.5

a Radiotherapy plus cetuximab.

**Table 2:** Patient and tumour characteristics, and associtation with fistula.

Characteristic	With fistula	Without fistula	*p*-Value
*Mean age*	65.7 ± 11.2	665 ± 1049	0.8

*Sex*			
Male	11 (44%)	14 (56%)	0.7
Female	1 (50%)	1 (50%)	

*Coronary artery disease*			
No	10 (43.5%)	13 (56.5%)	0.6
Yes	2 (50%)	2 (50%)	

*Chronic obstructive pulmonary disease*			
No	8 (42.1%)	11 (57.9%)	0.5
Yes	4 (50%)	4 (50%)	

*Hypertension*			
No	8 (47.1%)	9 (52.9%)	0.5
Yes	4 (40%)	6 (60%)	

*Diabetes mellitus*			
No	7 (36.8%)	12 (63.2%)	0.2
Yes	5 (62.5%)	3 (37.5%)	

*Chronic renal failure*			
No	11 (44%)	14 (6%)	0.2
Yes	1 (50%)	1 (50%)	

*Haemoglobin*			
>12 g/dL	5 (41.6%)	7 (58.4%)	0.6
<12 g/dL	7 (46.6%)	8 (53.4%)	

*Creatinin*			
<1.3 mg/dL	11 (47.8%)	12 (57.2%)	0.3
>1.3 mg/dL	1 (25%)	3 (75%)	

*Total proteins*			
>7 mg/dL	5 (38.5%)	8 (61.5%)	0.4
<7 mg/dL	7 (50%)	7 (50%)	

*Anesthetic assessment*			
ASA II	5 (28.5%)	8 (61.5%)	0.5
ASA III	4 (40%)	6 (60%)	
ASA IV	3 (75%)	1 (25%)	

*Chemotherapy*			
Yes	5 (41.6%)	7 (58.4%)	0.6
No	7 (46.6%)	8 (53.4%)	

*Tumour grade*			
Well	1 (25%)	3 (75%)	0.5
Moderately	7 (43.7%)	9 (56.3%)	
Poorly	4 (57.1%)	3 (42.9%)	

*Time until salvage laryngectomy*			
<12 months	4 (40%)	6 (60%)	0.5
>12 months	8 (47.1%)	9 (52.9%)	

*Localization*			
Supraglottis	6 (50%)	6 (50%)	0.4
Glottis	6 (40%)	9 (60%)	

*Tumour size*			
T1–T2	4 (36.4%)	7 (63.6%)	0.3
T3–T4	8 (50%)	8 (50%)	

*Node status*			
N0	11 (47.8%)	12 (52.2%)	0.3
N +	1 (25%)	3 (75%)	

*Tumour stage*			
I–II	4 (40%)	6 (60%)	0.5
III–IV	8 (47.1%)	9 (52.9%)	

*Prior tracheostomy*			
Yes	2 (20%)	8 (80%)	0.057
No	10 (58.8%)	7 (41.2%)	

*Neck dissection*			
Yes	2 (40%)	3 (60%)	0.6
No	10 (45.5%)	12 (54.5%)	

*Tracheoesophageal puncture*			
Yes	2 (40%)	3 (60%)	0.6
No	10 (45.5%)	12 (54.5%)

**Table 3:** PCF in patients with or without PMF interposition.

Variable	Without PMF (*n* = 14)	With PMP (*n* = 13)	*p*-Value
*Global PCF rate*			
Yes	11 (78.6%)	4 (30.8%)	0.016
No	3 (21.4%)	9 (69.2%)	

*PCF rate with surgical repair*			
Yes	9 (64.3%)	1 (7.7%)	0.003
No	5 (35.7%)	12 (92.3%)	

*Great pharyngostomas*			
Yes	9 (64.3%)	0 (0%)	0.0004
No	5 (35.5%)	13 (100%)	

PCF, pharyngocutaneous fistula.

**Table 4:** Surgical time, oral diet initiation and length of hospitalization.

FLAP	Mean	SD	Range	*p*-Value
Mean surgical time (minutes)				0.0075
With PMF (*n* = 13)	291.5	108.84	190–335	
Without PMF (*n* = 14)	199.28	45.81	165–280	

Mean surgical time including additional procedures to reconstruct pharyngostomes (minutes)				0.17
With PMF (*n* = 13)	299.23	110.33	190–340	
SIN Without PMF (*n* = 14)	385.21	192.58	165–985	

Oral diet initiation (days)				0.039
With PMF (*n* = 13)	21.53	14.64	7–61	
Without PMF (*n* = 14)	84	69.25	11–203	

Length of hospitalization (days)				0.0041
With PMF (*n* = 13)	27.23	14.48	10–63	
Without PMF (*n* = 14)	98.38	79.76	14–204	

## Discussion

Pharyngocutaneous fistula remains a common complication following salvage TL, with profound deleterious effects on wound healing, nutrition, hospitalization duration, initiation of oral diet, cost and quality of life. Meta-analysis of risk factors contributing to fistula include tumor stage, concurrent neck dissection, prior tracheostomy, anemia, hypoalbuminemia, surgical margins, and others.^6^, ^7^ Especially notable is the incidence, severity and duration of PCF in patients who received preoperative RT with or without chemotherapy.^12^ A meta-analysis of 25 studies by Paydarfar et al. demonstrates a 2.28 times increased risk of PCP among patients who received radiation or CRT prior to laryngectomy.^7^ Weber et al. published the outcomes of 129 patients in the RTOG 91–11 trial who needed STL, and the incidence of PCF was 30% in patients who underwent CRT, which was twice that seen in patients who failed radiation therapy alone.^13^ This is the usual percentage of fistula that is normally established in the radiated patient, although we believe that it is generally higher, as in our study, which reached 55%. In many cases, it also depends on what is considered PCF by surgical teams, if they include only large pharyngostomes requiring surgical repair or also small punctiform fistulas that usually resolve spontaneously with compression alone. There are studies that suggest that complications are more common when STL is performed within the first year after RT, and also if neck dissection is performed at the time of laryngectomy.^3^, ^14^ However, we did not see statistical differences between our patients where laryngectomy was performed in the first 12 months (60% vs. 52.9%, *p *> 0.05), nor in those where neck dissection was performed (60% vs. 54.5%, *p *> 0.05). However there is enough unanimity to consider that generally PCF's following CRT are more likely to need surgical repair than those that occur after primary total laryngectomy.^8^, ^13^, ^15^

Radiotherapy and CRT not only kill tumor cells but also cause damage to other tissues, including blood vessels. After irradiation increases of intima thickness, proteoglycan deposition, and inflammatory cell infiltrate in medium-sized arteries is observed normally resistant to natural atherosclerosis process.^16^ This explains the delay in healing and the greater formation of fistula. Several surgical strategies to minimize PCF following STL have been explored. Among those is the transfer of non-irradiated tissue in the form of a vascularized flap, which should aid wound healing and prevent fistula formation. The idea behind this concept is to reinforce the fragile suture line of the neopharynx and cover the previously irradiated soft tissue and mucosa with non-irradiated well-vascularized tissue.^11^

In broad terms, surgeons have adopted two distinct techniques when using vascularized tissue following laryngectomy. First, vascularized tissue is employed to augment the pharyngeal circumference by a patch graft. With this technique, several studies have demonstrated to have a lower rate of fluid leak.^17^, ^18^ Second, vascularized tissue to reinforce the pharyngeal repair. The idea is to reinforce the suture line by covering it with healthy muscle, anchored to the base of tongue, the pharyngeal constrictors, and the posterior wall of the trachea. Some studies using this technique have also observed lower fistula rate.^19^, ^20^

Since its first description in 1979, the pectoralis major muscle flap has become a standard technique for the reconstruction of head and neck defects. It can be designed incorporating a skin paddle, Pectoralis Major Myocutaneous Flap (PMMC), or only muscle and fascia, Pectoralis Major Myofascial Flap (PMMF). There are several advantages to using the PMF in this setting. The muscle flap is generally harvested while the pharynx is being closed and thus adds minimal time to the length of the operative case. Being a regional rotational flap, the pectoralis does not require a separate reconstructive team or microsurgical experience, and also no comprehensive flap monitoring is needed that might otherwise be indicated for free flaps. However, we must also take into account the pectoral muscle flap morbidity. There are some studies that reflect the shoulder dysfunction when this flap is harvested,^21^ negative consequences in the phonatory rehabilitation due the presence of muscle over the neopharynx, in addition to the cosmetic repercussions. Rarely, there may be difficulties in primary closure of the skin. We have not analyzed in our study the morbidity of the flap, although we believe that the advantages offered overcome the disadvantages.

Another controversy arises between using myocutaneous or myofascial flaps. There are several factors that may favor a muscle onlay technique with PMMF over an insetting technique with PMMC. Inserting a PMMC into the pharyngotomy can increase the length of the closure but also the potential length for fistula formation. A muscle onlay with PMMF reinforces the standard pharyngotomy closure with well vascularized fascia and muscle; perhaps, the vascularity of the pectoralis muscle may be more robust and reliable than the peripheral edges of the myocutaneous flap or a free flap, and thereby more capable of sealing off the pharyngotomy. Some studies reflect that the deep fascia surrounding the pectoralis muscle is rich in hyaluronan and can play an important role during earliest stages of wound healing.^22^, ^23^ We have used both types of flap. Our decision has depended mainly on the length of excess pharyngeal tissue: if the length was enough to close primarily with a suitable circumference then we used a myofascial flap; if by contrast we thought that the pharyngeal closure was going to be narrow, then we used a myocutaneous flap. Generally we prefer the muscle onlay technique because of its simplicity. Several studies have compared fistula rates between utilization of PMMC and PMMF. Khan et al.^24^ observed a leak rate of 26% in patients with myofascial flap, comparing to 33% in irradiated patients where a myocutaneous flap was used to reinforce the STL. Gilbert et al.^25^ compared PCF rate with primary closure (45%), myocutaneous flap (28.6%) and myofascial flap (10.5%), reaching similar results. In our study we have not seen statistically significant differences between using PMMC (25%) and PMMF (33%).

Other alternatives are free flaps, mainly the radial forearm or anterolateral thigh flap. With both there is a minimal functional morbidity and patient complaints are primary cosmetic. However, we must take into account that microvascular techniques add time and requires identification and preparation of donor vessels in a previously-radiated field. Also necessary are the microvascular expertise, equipment and flap monitoring, compared to the simplicity of the pectoralis major flap. A large multi-institutional study examined 359 patients, all with previous history of radiation, and the rate of fistula formation with primary closure vs. PMMF vs. interposed free flap.^12^ They found that the fistula rate was 34% with primary closure, 25% in the free flap group, and the lowest for the pectoralis onlay group at 15%.

Similar to other authors^12^ we did not identify other factors that are commonly associated with fistula. We did not see statistically significant differences in the rate of PCF in patients with low levels of hemoglobin or total protein. We also did not see association in patients where chemotherapy was added to radiotherapy, those with higher tumor stage or with concurrent neck dissection. One explanation for this is that the use of vascularized tissue may mitigate the impact of these other factors on fistula development. Interestingly, we found that the fistula rate was practically twice in those patients with prior tracheostomy (80% vs. 41.2%), without a result statistically significant (*p *= 0.057). This may be due to the fact that the STL is carried out in a tissue not only radiated but also previously operated.

There are two systematic reviews that justify the use of vascularized tissue in STL. Paleri et al.^26^ found that patients who have flap reconstruction/reinforcement reduced their risk of PCF by one-third. Similarly, Guimaraes et al.^27^ analyzed the efficacy of pectoralis major muscle flap for PCF prevention in salvage total laryngectomy, and observed that there was a 22% decreased risk of fistula incidence when the flap was used. In Table 5 we collect other studies. We can observe how, similarly to other authors, the PCF rate is much lower in patients with a prophylactic pectoralis major flap (78.6% vs. 30.7%). It is striking that in spite of the systematic use of a pectoral flap for years we still have fistula rates superior to other authors. This may be due to the fact that we have considered any leakage, not only the large pharyngostomes that require surgical repair but also the small punctate fistulas that usually resolve spontaneously. On the other hand, when we compare the fistulas that had to be reoperated, we have the same results as in other studies, with much lower fistula rates when we use the flap (64.3% vs. 7.7%).

**Table 5:** Data from other studies.

Reference/year	Pharyngocutaneous fistula		Surgical treatment of fistula	
	CMPM	N° CMPM	CMPM	N° CMPM
Righini et al., 2005	6/26 (23.1%)	17/34 (50%)	4/6 (66.6%)	14/17 (82.4%)
Gil et al., 2009	3/11 (27.3%)	16/89 (18%)	0/3	14/17 (82.4%)
Patel et al., 2009	0/10 (0%)	4/7 (57.1%)	–	–
Sakai et al., 2012	1/13 (77.7%)	12/39 (30.7%)	0/1 (0%)	10/12 (83.3%)
Sousa et al., 2012	3/19 (15.8%)	7/12 (58.3%)	–	–
Cömert et al., 2014	9/21 (42.8%)	14/24 (58.3%)	0/9 (0%)	10/14 (71.4%)
Sayles at al. 2014	6/13 (46.2%)	17/47 (36.2%)	–	–
Gilbert et al., 2014	6/35 (17.1%)	17/38 (44.7%)	–	–
Gendreau-Lefevre et al., 2014	7/51 (13.7%)	41/115 (35.6%)	4/7 (57.1%)	26/41 (63.41%)
Present study	4/13 (30.7%)	11/14 (78.6%)	1/4 (7.7%)	9/11 (64.3%)

Despite this, one can argue that potential morbidity because of raising and inserting the PMF largely outweighs the morbidity of a possible pharyngocutaneous fistula. We, however, think that the morbidity of the flap is minimal if we consider the possible consequences of a fistula for the patient. When this flap is not initially interposed, we have observed in our patients that posterior surgical reconstruction is usually laborious and often unsatisfactory. Even being a rare event, large pharyngostomes can produce wound breakdown and damage to nearby tissue and blood vessels, including carotid artery blowout, as we saw in one of our patients, and become a lethal complication. In our study we have observed that the oral diet initiation (84 days vs. 21.5 days) and the duration of hospitalization (98.3 days vs. 27.2 days) is much lower in patients with a preventive flap. This is beneficial especially for the patient but also in terms of economic costs. When we talk about larynx cancer we should not forget what the patient's preferences are.^28^ Patients should be informed about the cure rate of the different treatment options, and of course, the duration of the treatment. That is why we believe that the application of this policy is beneficial for both the surgeon and the patient. Although the prophylactic use of a PMF is not a routine in salvage total laryngectomy in most centers, we think that the impact of salivary leak on the morbidity, delay to oral diet initiation and hospital length of stay, justify the search for strategies to reduce its incidence.

The limitations of this study is that it is a retrospective study and not a double blind randomized clinical trial, in addition to the number of patients recruited because it is from a single institution. However, the results found in our patients are similar to those collected by other authors who recommend the preventive use of a PMF in patients undergoing TL previously irradiated. The main reasons we found to adopt this policy are: (1) Decrease in total number of pharyngocutaneous fistulas, of those that required surgical reintervention and large pharyngostomas; (2) surgical time is longer initially, however when we had not used PMF many patients had developed pharyngostomas that required complex reconstructions, and adding all those surgeries, on average the time in the operating room was longer; (3) less time to start oral intake; (4) shorter hospitalization time; (5) lower economic cost.

## Conclusions

Our study shows that pharyngocutaneous fistula remains a serious complication following salvage total laryngectomy. The pectoralis major flap is useful for improving the outcome of this procedure, promoting faster healing and preventing major complications. The incidence of fistula was lower in patients who underwent a flap compared to patients where it was not done. Both time until resumption of oral diet and length of hospitalization stay was shorter in patients with prophylactic flap. For all this we should recommend its use in all salvage total laryngectomies, although it is necessary to design and carry out a well-designed randomised-controlled trial to eliminate confounding factors and establish its usefulness.

## Conflicts of interest

The authors declare no conflicts of interest.
